# Evaluation of Alpha 1-Antitrypsin and the Levels of mRNA Expression of Matrix Metalloproteinase 7, Urokinase Type Plasminogen Activator Receptor and COX-2 for the Diagnosis of Colorectal Cancer

**DOI:** 10.1371/journal.pone.0051810

**Published:** 2013-01-02

**Authors:** Luis Bujanda, Cristina Sarasqueta, Angel Cosme, Elizabeth Hijona, José M. Enríquez-Navascués, Carlos Placer, Eloisa Villarreal, Marta Herreros-Villanueva, María D. Giraldez, Meritxell Gironella, Francesc Balaguer, Antoni Castells

**Affiliations:** 1 Department of Gastroenterology, Donostia Hospital-Biodonostia Institute, University of Basque Country (UPV/EHU), Centro de Investigación Biomédica en Enfermedades Hepáticas y Digestivas (CIBERehd), San Sebastián, Spain; 2 Department of Epidemiology, CIBERESP, Donostia Hospital, San Sebastián, Spain; 3 Department of Surgery, Donostia Hospital, San Sebastián, Spain; 4 Department of Gastroenterology, Clinic Hospital, CIBERehd, IDIBAPS, Barcelona, Spain; University of Bari & Consorzio Mario Negri Sud, Italy

## Abstract

**Background:**

Colorectal cancer (CRC) is the second most common cause of death from cancer in both men and women in the majority of developed countries. Molecular tests of blood could potentially provide this ideal screening tool.

**Aim:**

Our objective was to assess the usefulness of serum markers and mRNA expression levels in the diagnosis of CRC.

**Methods:**

In a prospective study, we measured mRNA expression levels of 13 markers (carbonic anhydrase, guanylyl cyclase C, plasminogen activator inhibitor, matrix metalloproteinase 7 (MMP7), urokinase-type plasminogen activator receptor (uPAR), urokinase-type plasminogen activator, survivin, tetranectin, vascular endothelial growth factor (VEGF), cytokeratin 20, thymidylate synthase, cyclooxygenase 2 (COX-2), and CD44) and three proteins in serum (alpha 1 antitrypsin, carcinoembryonic antigen (CEA) and activated C3 in 42 patients with CRC and 33 with normal colonoscopy results.

**Results:**

Alpha 1-antitrypsin was the serum marker that was most useful for CRC diagnosis (1.79±0.25 in the CRC group vs 1.27±0.25 in the control group, P<0.0005). The area under the ROC curve for alpha 1-antitrypsin was 0.88 (0.79–0.96). The mRNA expression levels of five markers were statistically different between CRC cases and controls: those for which the ROC area was over 75% were MMP7 (0.81) and tetranectin (0.80), COX-2 (0.78), uPAR (0.78) and carbonic anhydrase (0.77). The markers which identified early stage CRC (Stages I and II) were alpha 1-antitrypsin, uPAR, COX-2 and MMP7.

**Conclusions:**

Serum alpha 1-antitrypsin and the levels of mRNA expression of MMP7, COX-2 and uPAR have good diagnostic accuracy for CRC, even in the early stages.

## Introduction

Colorectal cancer (CRC) is a major cause of morbidity and mortality worldwide and is the second most common cause of cancer death in Europe and the United States [1], [2]. The overall five-year survival rate is less than 65% and when the cancer is already at an advanced stage when diagnosed treatment has very limited success. Currently, 25% of patients have metastasis at the time of diagnosis [3]. Detection of cancer at early stages is critical for curative treatment interventions. However, the invasive, unpleasant and inconvenient nature of current diagnostic procedures limits their applicability. Noninvasive molecular approaches would enable detection of relevant lesions at the population level and could be enhanced by widely distributable screening tools that are accurate, user-friendly, safe and affordable. Nonconventional methods could potentially meet all of the desired criteria of an ideal screening tool, so there is good reason to pursue rationally designed alternative approaches.

In particular, molecular tests of blood could potentially provide this ideal screening tool. However, the use of single or a combination of serum markers, including carcinoembryonic antigen (CEA), has so far failed to deliver diagnostic tests of high sensitivity and specificity for CRC.

There is reasonable hope and further evidence emerging that the presence of CRC could be detected by specific changes in the composition of serum proteins or mRNA expression levels [4]. Different markers are overexpressed in CRC compared with matched normal adjacent mucosa [5]. These observations suggest a model wherein as a key regulator of epithelial cell proliferation along the associated with neoplastic transformation. The targeting of RNA makes use of the fact that tumour phenotypic variation is associated with changes in the mRNA levels of genes regulating or effecting this variation. This has led to the widespread use of real-time reverse transcription polymerase chain reaction (RT-PCR) assays for the study of tumour dissemination as well as many reports describing its use in clinical diagnostics [6]. In addition, the levels of mRNA expression in blood for various proteins, in particular cyclooxygenase 2 (COX-2), vascular endothelial growth factor (VEGF) and others including metalloproteinase [7]–[9], have been proposed for CRC diagnosis and prognosis. Various different serum markers such as the complement protein C3a and alpha 1-antitrypsin have been judged to be useful in the diagnosis of CRC [10], [11]. Some of them, such as VEGF or urokinase-type plasminogen activator receptor (uPAR) have been studied in other intestinal diseases such as inflammatory bowel disease and have not found elevated [12], [13]. Other markers, such as tetranectin were found elevated in other gastrointestinal and gynecological tumors [14], [15].

Our objective in this study was to assess the usefulness of different serum and mRNA expression markers for the diagnosis of CRC.

## Materials and Methods

Sixteen serum markers were studied prospectively in 42 patients with CRC and 33 healthy controls. Individuals with CRC who had inflammatory bowel disease and polyps were excluded. Participants with CRC were staged according to the 5^th^ edition of the TNM classification [16].

The controls were individuals with no signs or symptoms of chronic disease and who had undergone a colonoscopy to the caecum with normal results. These controls were not taking drugs or NSAIDs. For all participants the following data was collected: age, weight, height, body mass index (BMI), associated medical conditions and medications taken.

The serum markers measured were alpha 1-antitrypsin, complement protein C3a and CEA. The markers in the blood analysed by RNA extraction, cDNA synthesis and quantitative PCR (qPCR) were carbonic anhydrase, guanylyl cyclase C, plasminogen activator inhibitor (PAI), matrix metalloproteinase 7 (MMP7), uPAR, urokinase-type plasminogen activator (uPA), survivin, tetranectin, VEGF, cytokeratin 20, thymidylate synthase, COX-2 and CD44. These last markers were determined in tumoral tissue and healthy tissue obtained after surgical resection of CRC. All markers were selected based on their relationship to diagnosis or prognosis in CRC after a review of the literature (Table S1).

For each participant, two fasting blood samples were taken from the antecubital vein. These blood tests were carried out in the 24 hours prior to surgery in the case of the patients diagnosed with CRC and 24 hours following the colonoscopy in the controls.

One of these samples was used to determine three proteins in serum (alpha 1-antitrypsin, activated C3 and CEA levels), while mRNA was extracted from the other. Blood samples from all participants were collected in PAXgene RNA blood tubes (QIAGEN, K2E 18.0 mg – BD, Plymouth, UK) and kept frozen at −80°C until RNA extractions were carried out.

### Measurement of Alpha 1-antitrypsin, Activated C3 and CEA Levels

The level of CEA in the serum was determined using an electrochemiluminescence immunoassay. Pre-operative levels of serum CEA of ≤5.0 ng/mL were considered normal.

Measurements of the serum concentration of complement C3a were performed using the OptEIA Human C3a ELISA kit (BD Biosciences Pharmingen, San Diego, CA, USA). Normal levels for serum complement C3a was taken to be between 10 mg/dl and 40 mg/dl.

Alpha 1-antitrypsin (g/l) concentrations were determined using an enzyme immunoassay kit (Dade Behring, Newark, NJ, USA) on a BN II ProSpec nephelometer. A cut-off level of 1.5 g/L was chosen as the upper limit of normal.

### RNA Extraction and cDNA Synthesis

Total RNA from 2.5 ml whole blood collected in the PAXgene blood RNA tubes was extracted using PAXgene Blood Minikit (QIAGEN, PreAnalytix cat: 762164) according to manufacturer’s instructions. Each RNA sample was quantified and quality assessed using an Agilent 2100 bioanalyzer system to obtain an RIN value. The RIN algorithm allows RNA integrity to be calculated using a trained artificial neural network based on the determination of the most informative features that can be extracted from the electrophoretic traces out of 100 features identified through signal analysis {IMBEAUD, 2005 #42}.

Total RNA from 20–30 mg of tissue (tumoral and healthy) was extracted using the RNeasy Plus Mini Kit (QIAGEN) following manufactureŕs instructions. This kit includes a DNase step which removes contaminant genomic material from RNA extractions. Using µg of RNA.

cDNA was synthesized by reverse transcription using a Superscript III kit (Invitrogen Ltd., Paisley, UK) following manufacturer’s instructions in a total volume of 20 µl. Subsequently cDNA was prepared from 1 µg of RNA using the Superscript III RT PCR kit (Invitrogen).

### Quantitative PCR (qPCR)

The qPCR was performed using a Bio-Rad iQ5 system with a total of 13 CRC potential markers (Table 1). Each qPCR reaction contained a gene-specific oligonucleotide primer set (25 uM), 12.5 µl Platinum qPCR supermix (Invitrogen) and 1/20 of the original cDNA reaction mix. Each reaction was carried out in duplicate, together with a negative control (H_2_O as template). PCR efficiency was assessed by performing triplicates of 5-fold serial dilutions from a pool of cDNA derived from 12 healthy individuals to obtain an R^2^ value of >0.98. To compensate for RNA degradation and variability in the starting amounts of RNA, four housekeeping genes, chosen with the GenEx Light Software (TATAA Biocenter, Sweden) from a panel of 13 genes, were used as endogenous controls (GAPDH, HPRT1, PPIA and TBP).

**Table 1:** Oligonucleotide primer sequences generated used for the detection of mRNA for CRC markers.

CRC Marker	NCBI Accession number	PrimerName	Primers sequence (5′-3′)	ProductSize (bp)
Carbonic anhydrase I/II	M33987	CAIF9CAIR10	AGCTACAGGCTCTTTCAGTTTCAT CTTCATCAAAACACCAATAACAGC	189
CD44	48255934	CDFCDR	AACACACCAGTGTCTGTTCTTGAT GACTGATGAATAAATGCCACAAAG	209
Cytokeratin-20	27894336	CKF9CKR10	CTGAATAAGGTCTTTGATGACC ATGCTTGTGTAGGCCATCGA	129
COX-2	AY462100.1	CoxF9CoxR10	TGCCTGGTCTGATGATGTATGCCA CTGCTTGTCTGGAACAACTGCTCA	115
Guanylyl Cyclase C	3702146	GCC5GCC6	TTACCAACAAGGAACAAACTCAAA GGTTGCTAATCTATTCCTGATGCT	102
MMP-7	35802	MMPF3MMPR4	ACATCATGATTGGCTTTGCGCGAG TCCCATACCCAAAGAATGGCCAAG	212
PAI-1	35275	PAIFPAIR	CTCATCCACAGCTGTCATAGTCTC CTTTTCTTCGGAGTTTCTTCTTTC	205
Survivin	56693630	SurvFSurvR	CCACCGCATCTCTACATTCA TATGTTCCTCTATGGGGTCG	185
Tetranectin	X64559	TetFTetR	GTGAACACAAAGATGTTTGAGGAG GTACTCATACAGGGCGTCGTTCT	246
Thymidylate synthase	NM_001071	TSFTSR	GCCTCGGTGTGCCTTTCA CCCGTGATGTGCGCAAT	67
uPA	NM_002658.2	uPA3uPA4	CGTCTACACGAGAGTCTCACACTT ACTCTACTGCAAAAATGACAACCA	140
uPAR	517197	URFURR	TGAAGAGACTTTCCTCATTGACTG CCACTTTTAGTACAGCAGGAGACA	189
VEGF	AY766116	VEGFVEGR	AGTGGTCCCAGGCTGCAC TCCATGAACTTCACCACTTCGT	70

The amplification values obtained from each of the 13 markers used in this analysis were divided by the corresponding amplification values for each of the housekeeping genes to produce an expression index. Of these, GAPDH produced the most stable results and was used in further analysis.

To determine changes in levels of expression of all the 13 markers in relation to the housekeeping genes we used the comparative ΔC_T_ method where the copy number of the marker gene (target) in the samples from patients with CRC (tumour) is compared to the relative amount of the target gene in normal cDNA from either its corresponding paired-sample or pooled blood samples and relative to expression of the housekeeping gene, which was used as an endogenous control (reference).

All CRC markers used in this study and their corresponding oligonucleotide primer sequences are listed in Table 1.

The committee for clinical research ethics committee of Country Basque approved this study and all participants gave written informed consent.

### Statistical Analysis

The comparison of the parameters analysed between CRC cases and controls were carried out using the Student’s t-test. In addition, ROC (Receiving Operating Characteristic) curve analysis, with calculation of both the area under the curve and the corresponding 95% confidence intervals, was used to assess the accuracy with which the parameters diagnosed CRC, that is, whether they could be used to discriminate between patients with CRC and controls. To evaluate the differences in some markers according to the stage has been applied univariate generalized linear model which has assessed linear trend by polynomial contrasts and multiple comparisons (post-hoc) pairwise Tukey’s test. Analyses were performed by using the SPSS statistical software, version 19 and MedCalc version 6.

## Results

### Subjects

Of the 42 patients with CRC 25 (59%) were men and 17 (41%) women. Their mean age was 72.33 years old and their BMI was 26.57. The tumours were classed as Stage I in 8 patients (20%), Stage II in 14 (33%), Stage III in 17 (40%) and Stage IV in 3 patients (7%).

Of the 33 controls, 20 (60%) were men and 13 (40%) were women, their mean age being 75 years old and BMI 26. There were no significant differences between the cases and controls in terms of age, sex or BMI.

When serum data were analysed, there were significant differences between cases and controls in the levels of alpha 1-antitrypsin. The alpha 1-antitrypsin levels were 1.79±0.25 in the patients with CRC compared to 1.27±0.4 in the controls (p<0.0005). CEA in serum was 1.5±1 in controls and 8.5±15 in cases (p = 0.01). However, differences in complement protein C3a concentrations between cases and controls were not significant (14.56±4.08 vs 12.89±6.5; p = 0.28).

mRNA expression levels of survivin, MMP7, uPA, COX-2 and CD44 were increased statistically significant in tissue with CCR versus normal tissue whereas carbonic anhydrase, tetranectin, and cytokeratin 20 were significantly decreased (Table 2).

**Table 2:** The levels of mRNA expression of the proteins analysed and the statistical significance of differences in the values between cancer tissue and healthy tissue in patients with CRC.

	TumoralMeanΔCt(Mean Ct)±SD	HealthyMeanΔCt(Mean Ct)±SD	Paired differencesAverage	*p* value
Carbonic anhydrase	9,02(29.5)±4.3	0,35(20.9)±2.5	8.61	0.000
Guanylyl cyclase C	6.90(27.4)±2.5	6.61(27.2)±1.1	0.15	0.06
PAI-1	10.1(28.9)±2.3	11.5(31.2)±2.6	2.20	0.02
MMP7	5.58(25.6)±2.1	10.09(30.7)±3.7	5.07	0.000
Survivin	6.21(26.2)±1.1	6.88(27.1)±0.7	0.8	0.03
uPAR	9.84(29.6)±2.3	9.33(29.9)±2.8	0.29	0.5
Tetranectin	8.68(29.8)±2.9	4.78(25.8)±1.3	3.96	0.000
uPA	6.34(24.4)±1.3	8.58(26.1)±3.6	1.72	0.005
VEGF	8.27(29.4)±2.1	9.31(30.4)±1.7	0.98	0.06
Cytokeratin 20	6.62(27.3)±2.3	2.58(23.2)±1.0	4.02	0.000
Thymidylate synthase	6.19(25.3)±1.0	6.16(25.1)±0.7	0.14	0.9
COX-2	7.45(26.6)±1.9	8.79(27.8)±1.5	1.28	0.002
CD44	12.67(32.5)±2.0	14.37(33.9)±1.1	1.44	0.01

SD; standard deviation PAI; plasminogen activator inhibitor MMP7;matrix metalloproteinase 7 uPAR; urokinase-type plasminogen activator receptor uPA;urokinase-type plasminogen activator VEGF; vascular endothelial growth factor COX-2; cyclooxygenase 2.

The mRNA expression levels in blood are reported in Table 3. Among these, there were five markers in which the differences between cases and controls were significant, namely, carbonic anhydrase, MMP7, uPAR, tetranectin, and COX-2. There were no significant differences in any of the 16 markers studied according to sex.

**Table 3:** The levels of mRNA expression in blood of the proteins analysed and the statistical significance of differences in the values between patients with CRC and controls.

	PatientsMean ΔCt±SD(Mean Ct)	ControlsMean ΔCt±SD(Mean Ct)	*p* value
Carbonic anhydrase	2.14(22.8)±3.6	4.46(22.6)±1.3	0.02
Guanylyl cyclase C	7.42(28.5)±2.9	7.54(26.4)±4.7	0.06
PAI-1	14.96(35.2)±2.8	16.06(34.2)±2.0	0.19
MMP7	15.51(35.2)±1.6	17.46(35.2)±1.3	0.001
Survivin	9.30(31.3)±3.3	10.34(29.9)±1.0	0.26
uPAR	8.77(30.8)±2.9	6.92(25.1)±0.8	0.004
Tetranectin	13.39(33.8)±1.2	11.34(29.1)±3.7	0.007
uPA	9.28(29.7)±3.6	10.10(27.7)±1.5	0.98
VEGF	9.85(30.8)±4.4	10.99(28.2)±1.6	0.83
Cytokeratin 20	14.08(34.4)±3.3	14.55(32.7)±2.2	0.6
Thymidylate synthase	8.44(28.3)±1.4	8.44(26.0)±1.0	0.9
COX-2	6.86(26.2)±1.1	5.91(23.6)±0.5	0.02
CD44	10.87(28.3)±4.3	10.62(27.4)±1.4	0.3

SD; standard deviation PAI; plasminogen activator inhibitor MMP7;matrix metalloproteinase 7 uPAR; urokinase-type plasminogen activator receptor uPA;urokinase-type plasminogen activator VEGF; vascular endothelial growth factor COX-2; cyclooxygenase 2.

In the case of the two serum markers that showed significant differences, the areas under the ROC curve were 0.88 (0.7–0.9) for alpha 1-antitrypsin and 0.84 (0.6–0.9) for CEA.

**Figure 1:**
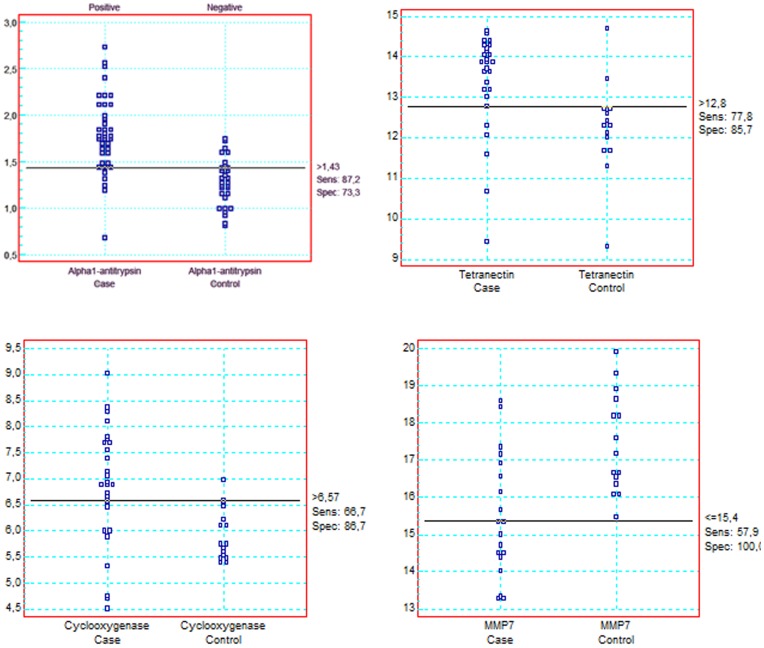
The results shown are the levels of alpha1 antitrypsin, tetranectin, COX-2 and matrix metalloproteinase 7 (MMP7) for the healthy individuals and patients with colorectal cancer. The cutoff corresponds with highest accuracy (minimal false negative and false positive). Shows the sensitivity and specificity for the cutoff with highest accuracy.

**Figure 2:**
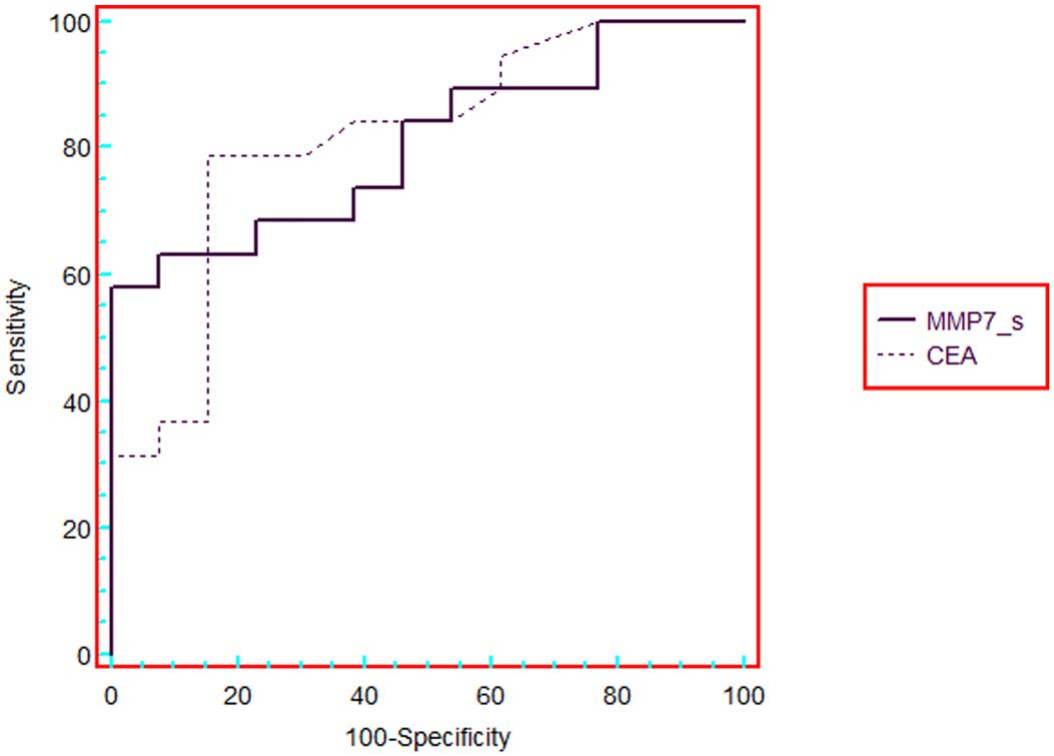
ROC curves comparing carcinoembrionic antigen (CEA) in serum and MMP7 RNA expression. The areas under ROC curve for CEA and matrix metalloproteinase 7 (MMP7) were 0.84 and 0.81, respectively.

For the mRNA expression, the areas under the ROC curve for the different markers are listed in Table 4. The highest diagnostic accuracies were found for MMP7 (0.81) and tetranectin (0.80), COX-2 (0.78), uPAR (0.78) and carbonic anhydrase (0.77) (Table 4). For screening, a context in which the priority is sensitivity and minimisation of false negatives, a suitable cut-off was 1.43 for alpha 1 antitrypsin, giving a sensitivity of 87% and a specificity of 73%. A value for MMP7 of 15.4 was the cut-off that minimised the percentage of false negatives, giving a sensitivity of 58% and specificity of 100% (Figure 1). In Figure 2 compares the area under the ROC curve between serum CEA and mRNA expression of MMP7.

**Table 4:** The areas under ROC curve for all the mRNA expression levels analysed in blood.

	Area under the ROC curve
Carbonic anhydrase	0.77 (0.6–0.9)
Guanylyl cyclase C	0.63 (0.5–0.8)
PAI-1	0.66 (0.5–0.8)
MMP7	0.81 (0.7–0.9)
Survivin	0.60 (0.4–0.8)
uPAR	0.78 (0.6–0.9)
Tetranectin	0.80 (0.6–0.9)
uPA	0.53 (0.4–0.7)
VEGF	0.54 (0.4–0.7)
Cytokeratin 20	0.50 (0.3–0.6)
Thymidylate synthase	0.52 (0.4–0.7)
COX-2	0.78 (0.6–0.9)
CD44	0.72 (0.6–0.8)

PAI; plasminogen activator inhibitor MMP7;matrix metalloproteinase 7 uPAR; urokinase-type plasminogen activator receptor uPA;urokinase-type plasminogen activator VEGF; vascular endothelial growth factor COX-2; cyclooxygenase 2.

When the values of the markers were compared between controls and patients with Stage I or II CRC, it was observed that alpha 1-antitrypsin, uPAR, COX-2 and MMP7 levels were significantly different, indicating that these compounds could be useful for the early detection of CRC (Table 5). By contrast, differences between groups in CEA and tetranectin were only significant for advanced tumours (Stages III and IV), indicating that are not suitable for early diagnosis of CRC.

**Table 5:** The mean levels of serum markers and mRNA expression in blood in the control group, patients with early stage (Stages I and II) and with advanced stage (Stages III and IV) CRC.

	AControlMean±SD	BStage I–IIMean±SD	CStage III–IVMean±SD	A vs B	A vs C	*p* value(Linear trendline)
Alpha1-antitrypsin	1.3**±**0.3	1.8**±**0.3	1.7**±**0.5	<0.0005	<0.0005	<0.0005
CEA	1.6**±**1.0	6.4**±**10.6	9.8**±**19.2	NS	0.04	0.01
Complement C3a	12.9**±**4.1	15.7**±**6.5	13.2**±**6.6	NS	NS	NS
MMP7	17.5**±**1.3	15.5**±**1.8	15.4**±**1.6	0.01	0.02	0.005
uPAR	6.9**±**0.8	10.1**±**3.0	7.7**±**2.8	0.003	NS	NS
Tetranectin	11.3±1.2	12.9±1.2	13.7±1.3	NS	0.04	0.01
COX-2	5.9±0.5	7.0±1.4	6.8±0.9	0.03	NS	0.02
CD44	10.6±1.4	11.4±4.5	10.4±4.2	NS	NS	NS

NS; Not significant SD; standard deviation uPAR; urokinase-type plasminogen activator receptor VEGF; vascular endothelial growth factor.

COX-2; cyclooxygenase 2 CEA; carcinoembryonic antigen MMP7;matrix metalloproteinase 7.

## Discussion

CRC has several the characteristics to justify population-wide screening: high prevalence, the occurrence of precancerous lesions, and the existence of treatment that is effective when applied in the early stages. The heterogeneity of colorectal neoplasms must be taken into account in selecting markers for molecular detection and determining the frequency of screening tests. Detection of these markers in blood depends on a cascade of biological events. Markers must be regularly released from tumours or precancerous lesions, dispersed into the medium that is collected for the assay, survive metabolic degradation, and be measurable [4]. In our study we analyzed 16 potential blood markers and watched as three may be useful in identifying early-stage CRC (uPAR, MMP7, COX-2 and alfa 1 antitrypsin). The combination of them could be useful for screening of CRC. Two of the 3 markers are mRNA expression determined by real-time or quantitative PCR (qPCR). This simply method is a variation of the standard PCR technique used to quantify mRNA in a sample. Using specific sequence primers, it is possible to determine the number of copies or relative quantity of given sequences of RNA. When real-time PCR is combined with reverse-transcription (sometimes called qRT-PCR), the quantity of mRNA in a sample can be ascertained for different proteins [6]. With this approach, it has been observed, for instance, that CEA mRNA expression in blood can predict recurrence in gastric cancer [17], esophageal squamous cell carcinoma [18] and CRC [19].

Soluble tetranectin was measured preoperatively in serum from patients with primary colorectal cancer. Tetranectin is a recently described human plasma protein, which is found in most secretory cells throughout the body and has been found to be a strong prognostic factor in patients with CRC in others studies [20]. Specifically, significant correlations were found between tetranectin and Dukes’ stages and other biochemical markers, such as PAI, uPAR and CEA. However, there are no previous reports of studies examining the tetranectin mRNA expression for CRC diagnosis. In our study, it was found that the expression of tetranectin mRNA had a good sensitivity and specificity (78% and 86%, respectively) to a cutoff of 12,8.

There is considerable experimental evidence that uPAR is functionally involved in cancer invasion, consistent with its ability to concentrate and enhance uPAR activity on the cell surface [21]. The level of the uPAR is elevated in tumour tissue from several forms of cancer [22]. It is known that uPAR is shed from the cell surface and the soluble form uPAR has been detected in several body fluids. The preoperative plasma uPAR level independently was found to predict survival of patients with CRC. In that study, the plasma concentration of uPAR was measured by a kinetic ELISA method in EDTA-anticoagulated plasma and not by level of expression of mRNA in serum as in our study. In colon cancer uPAR mRNA expression is enhanced in tumour cells [23]. In our study the expression on blood showed significant differences with controls y entre controles y pacientes con estadios I-II de CCR.

Other studies have demonstrated how mRNA expression levels of angiogenic factors, such as VEGF, in liver metastasis can be predicted from those in primary colorectal cancer [7]. Further, the measurement of thymidylate synthetase mRNA in primary colorectal cancer can reflect those in synchronous liver metastases [24]. However, most studies have investigated the levels of mRNA expression in tissue but not in blood as we have. We did not observe differences in thymidylate synthetase and VEGF in tissue or blood of patients with CRC. Is possible that there were not differences due to the small number of patients with stage IV.

Proteomic profiling of serum from patients with CRC and controls combined with the use of artificial neural networks was able to diagnose CRC with 94% sensitivity and 96% specificity in a cohort of patients. Using surface-enhanced laser desorption/ionisation (SELDI), Ward et al. in 2006 [25] identified four proteins (alpha 1-antitrypsin, complement C3a, transferrin and apolipoprotein C1) potentially useful for the diagnosis of CRC. The proteins identified are common serum proteins and changes in their concentrations most likely reflect epiphenomena rather than secretion by cancer cells. In that study (24), CEA was measured in all of the samples (62 CRC patients and 31 subjects not diagnosed with cancer), and a using the manufacture’s recommended cut-off level of 4 ng/ml, the sensitivity and specificity obtained was 53% and 93%, respectively. By contrast, in our study, though we also studied the levels of transferrin we did not find significant differences between patients with CRC and controls, (225.4±53.69 vs 231.1±37.1; p = 0.60) with an area under the ROC curve of 0.47 (0.34 to 0.61). Levels of apolipoprotein C1, on the other hand, were not investigated. CEA is clinically used as marker of CRC progression, but alone is not useful as a marker diagnostic [26]. In our study, the ROC values was higher to others studies [27], but was not useful in differentiating early stages and a control group. Unlike other studies, the combination with other markers did not improve the ROC curve.

Elevated serum alpha 1-antitrypsin levels have been observed in association with malignancy and inflammation. Some authors [10], [26] have shown that serum alpha 1-antitrypsin levels correlated with CRC and with clinical staging. In these studies, the correlation of CEA and alpha 1-antitrypsin to the stage of disease had almost the same statistical weight (p<0.004 and p<0.003)(26). The other three markers (C-reactive protein, alpha 1-acid glycoprotein and the percentage of serum protein electrophoretic components) in the series of Stamatiadis et al. [26] had a less significant correlation to the spread of the disease. Nevertheless, their potential role in the diagnosis of CRC, especially in comparison with other mRNA expression markers had not been established until now. Interestingly, high levels of alpha 1-antitrypsin in gastric juice have been associated with gastric cancer [27], [28]. In our study we observed that serum levels of alpha 1-antitrypsin were increased compared to controls. Serum levels were increased by stage and discriminated between early and advanced stages.

Complement C3a is highly biologically active, binding to mast cells and basophils and triggering the release of their vasoactive contents. The elevated level of complement C3a in the serum of CRC patients may reflect an immune response to the tumour, or possibly in vitro complement activation [29]. Based on a specific ELISA, serum levels of complement C3a predicted the presence of CRC in a blinded validation set with a sensitivity of 96.8% and a specificity of 96.2% [11]. Increased serum levels were also detected in 86.1% of independently collected sera from patients with colorectal adenomas, while only 5.6% were classified as normal. In our study it was not useful to discriminate cases and controls.

The discrepancies observed in some markers in our study between tissue and blood may be due to different reasons, first, not all mRNA is overexpressed in tumor tissue enter in blood. Second, the mRNA extracted from whole blood comes from blood cells (leukocytes, platelets …), very different from mRNA extracted from CCR. Third, the blood carries RNases (enzymes that degrade RNA), so that RNA circulating in the blood is more likely to be degraded. This is more difficult to occur in the RNA extracted from tumor cells.

Among the limitations of the study are; first, the small sample size; second, unknown if the results well be reproduced in asymptomatic individuals over 50 years (screening); finally, we also unknown whether the expression of these markers in patients with other gastrointestinal diseases such as inflammatory bowel disease, may be altered. Other studies are needed in other diseases and other gastrointestinal tumors to determine the value of these markers in these diseases. For example, tetranectin has not been studied in inflammatory bowel disease but has been elevated in other tumors such as gynecological, gastric, etc.

In summary, we report that alpha 1-antitrypsin and uPAR, COX-2 and MMP7 mRNA expression in blood could be useful for the early diagnosis and/or screening of CRC. Further studies in other cohorts the patients and screening programs are required involving larger numbers of subjects to confirm these results.

## Supporting Information

Table S1 mRNA expression selected, samples where were determined, application in colorectal cancer and references that support
(DOC)
