# Supplementary material for: Evaluation of Alpha 1-Antitrypsin and the Levels of mRNA Expression of Matrix Metalloproteinase 7, Urokinase Type Plasminogen Activator Receptor and COX-2 for the Diagnosis of Colorectal Cancer
Source: PLoS One. 2013 Jan 2;8(1):e51810. doi: 10.1371/journal.pone.0051810 (PMC3534697; doi:10.1371/journal.pone.0051810)
Supplement: Table S1 — mRNA expression selected, samples where were determined, application in colorectal cancer and references that support (DOC) [file pone.0051810.s001.doc]

**Supplementary Table 1.** Messenger RNA expression selected, samples where were determined, application in colorectal cancer and references that support

| **Biomarker** | **Source** | **Application in CRC** | **References** |
| --- | --- | --- | --- |
| Guanylyl cyclase C (GCC) | RNA from tissue | Diagnosis - Prognosis | Birbe *et al.*, 2005; Buc *et al.*, 2005; Frick *et al.*, 2005 |
| Cytokeratin 20 (CK20) | RNA from tissue and blood | Diagnosis - Stage – Prognosis | Chen and Wang, 2004; Lassmann *et al.*, 2004; McGregor *et al.*, 2004 ; Conzelmann *et al.*, 2005; Dandachi *et al.*, 2005; Iinuma *et al.*, 2006; Yeh *et al.*, 2006 |
| Vascular endothelial growth factor (VEGF) | RNA from tissue | Diagnosis - Prognosis | Furodoi *et al.*, 2002 ; White *et al.*, 2002; Hanrahan *et al.*, 2003 ; Galizia *et al.*, 2004 ; Ferroni *et al.*, 2005 |
| CD44 | RNA from tissue | Prognosis | Clarke *et al.*, 2000; Vizoso *et al.*, 2004 ; Zavrides *et al.*, 2005 ; |
| Thymidylate synthase | RNA from tissue | Therapy - Prognosis | Graziano *et al.*, 2003; Bendardarf *et al.*, 2004; Libra *et al.*, 2004; Popat *et al.*, 2004; Valbohmer *et al.*, 2006; |
| Urokinase Plasminogen Activator Receptor (uPAR) | RNA from tissue | Prognosis | Graziano *et al.*, 2003; Terada *et al.*, 2005 |
| Plasminogen Activator Inhibitor-1 (PAI-1) | RNA from tissue | Prognosis | Sakakibara *et al.*,  2005; Terada *et al.*, 2005 |
| Urokinase Plasminogen Activator uPA | RNA from tissue and blood | Diagnosis - Stage – Prognosis | Graziano *et al.*, 2003 |
| Cyclooxygenase-2 (Cox-2) | RNA from tissue | Prognosis | Garcea *et al.*, 2003; Soumaoro *et al.*, 2004 ; Yao *et al.*, 2005 |
| Survivin | RNA from tissue | Prognosis | Sela, 2002; Yao *et al.*, 2004; Suga *et al.*, 2005 |
| Matrix metalloproteinases (MMP) | RNA from tissue | Diagnosis | Bendardarf *et al.*, 2004 |
| Tetranectin | RNA from tissue | Prognosis | Notterman *et al.*, 2001; Birkenkamp-Demtroder *et al.*, 2002; Hodgall *et al.*, 2002 |
| Carbonic anhydrase | RNA from tissue | Prognosis | Bekku *et al.*, 2000; Kitahara *et al.*, 2001; Notterman *et al.*, 2001; Birkenkamp-Demtroder *et al.*, 2002 ; Robertson *et al.*, 2004 |

**References of supplementary table 1.**

1. **Bekku, S., Mochizuki, H., Yamamoto, T., Ueno, H., Takayama, E., Tadakuma, T.** (2000). Expression of carbonic anhydrase I or II and correlation to clinical aspects of colorectal cancer. *Hepatogastroenetrology.* **47 (34)**, 998-1001.
2. **Bendardaf, R., Lamlum, H., Pyrhonen, S.** (2004). Prognostic and predictive molecular markers in colorectal carcinoma. *Anticancer Res.* **24 (4),** 2519-30.
3. **Birbe, R., Palazzo, J.P., Walters, R., Weinberg, D., Schutz, S. Waldman, S.A.** (2005). Guanylyl cyclase C is a marker of intestinal metaplasia, dysplasia, and adenocarcinoma of the gastrointestinal tract. *Human Pathology.* **36 (2)**, 170-79.
4. **Birkenkamp-Demtroder, K., Christensen, L.L., Olesen, S.H., Frederiksen, C.M., Laiho, P., Aaltonen, L.A., Laurberg, S., Sorensen, F.B., Hagemann, R., Orntoft, T.F.** (2002). Gene expression in colorectal cancer. *Cancer Research.* **62**, 4352-63.
5. **Buc, E., Vartanian, M.D., Darcha, C., Dechelotte, P., Pezet, D.** (2005) Guanylyl cyclase Cas a reliable immunohistochemical marker and its ligand Escherichi coli heat-stable enterotoxin as a potential protein-delivering vehicle for colorectal cancer cells. *Eur.* *Journal Cancer*. **41 (11)**, 1618-1627.
6. **Chen, Z.M.E., Wang, H.L.L.** (2004). Alteration of cytokeratin 7 and cytokeratin 20 expression profile is uniquely associated with tumorigenesis of primary adenocarcinoma of the small intestine. *American Journal of Surgical Pathology.* **28 (10),** 1352-59.
7. **Clarke, G., Ryan, E., O’Keane, J.C., Crowe J., Mathuna, P.M.** (2000). Mortality association of enhanced CD44v6 expression is not mediated through occult lymphatic spread in stage II colorectal cancer. *J. Gastroenterol. Hepatol.* **15 (9),** 1028-31.
8. **Conzelmann, M., Linnemann, U., Berger. M.R.** (2005). Molecular detection of clinical colorectal cancer metastasis: how should multiple markers be put to use? *International Journal of Colorectal disease*. **20 (2)**, 137-146
9. **Dandachi, N., Balic, M., Stanzer, S., Halm, M., Resel M., Hinterleitner, T.A., Samonigg H., Bauernhofer T.** (2005). Critical evaluation of real-time reverse transcriptase-polymerase chain reaction for the quantitative detection of cytokeratin 20 mRNA in colorectal cancer patients. *Journal of Molecular Diagnostics*. **7 (5)**, 631-637.
10. **Ferroni, P., Spila, A., Martini, F., D’Alessandro, R., Mariotti, S., Del Monte, G., Graziano, P., Buonomo, O., Guadagni, F., Roselli, M.** (2005). Prognostic value of vascular endothelial growth factor tumor tissue content of colorectal cancer. *Oncology.* **69 (2),** 145-53.
11. **Frick, G.S., Pitari, G.M., Weinberg, D.S., Hyslop, T., Schulz, S., Waldman, S.A.** (2005). Guanylyl cyclase C: a molecular marker for staging and postoperative surveillance of patients with colorectal cancer. *Expert review of molecular diagnostics.* **5 (5)**, 701-713.
12. **Furodoi, A., Tanaka, S., Haruma, K., Kitadai, Y., Yoshihara, M., Chayama, K., Shimamoto, F.** (2002). Clinical significance of vascular endothelial growth factor C expression and angiogenesis at the deepest invasive site of advanced colorectal carcinoma. *Oncology.* **62 (2)**, 157-66.
13. **Galizia, G., Lieto, E., Ferraraccio, F., Orditura, M., De Vita, F., Castellano, C., Ciardiello, F., Agostini, B., Pignatelli, C.** (2004). Determination of molecular marker expression can predict clinical outcome in colon carcinomas. *Clinical Cancer Research.* **10**, 3490-99.
14. **Garcea, G., Sharma, R.A., Dennison, A., Steward, W.P., Gescher, A., Berry, D.P.** (2003). Molecular biomarkers of colorectal carcinogenesis and their role in surveillance and early intervention. *European Journal of Cancer.* **39 (8)**, 1041-52.
15. **Graziano, F. and Cascinu, S.** (2003). Prognostic molecular markers for planning adjuvant chemotherapy trials in Duke´s B colorectal cancer patients: how much evidence is enough? *Ann Oncol.* **14(7),** 1026-38.
16. **Hanrahan, V., Currie, M.J., Cunningham S.P., Morrin, H.R., Scott, P.A., Robonson, B.A., Fox, S.B.** (2003). The angiogenic switch for vascular endothelial growth factor (VEGF)-A, VEGF-B, VEGF-C, and VEGF-D in the adenoma-carcinoma sequence during colorectal cancer progression. *Journal of Pathology.* **200 (2),** 183-93.
17. **Hogdall, C.K., Christensen, I.J., Stephens, R.W., Sorensen, S., Norgaard-Pedersen, B., Nielsen, H.J.** (2002). Serum tetranectin is an independent prognostic marker in colorectal cancer and weakly correlated with plasma suPAR, plasma PAI-1 and serum CEA. *APMIS.* **110 (9)**, 630-38.
18. **Iinuma, H., Okinaga, K., Egami, H., Mimori, K., Hayashi, N., Nishida, K., Adachi, M., Mori, M., Sasako, M.** (2006) Usefulness and clinical significance of quantitative real-time RT-PCR to detect isolated tumor cells in the peripheral blood and tumor drainage blood of patients with colorectal cancer. *Int J Oncol.* **28 (2)**, 297-306.
19. **Kitahara, O., Furakawa, Y., Tanaka, T., Kihara, C., Ono, K., Yanagawa, R., Nita, M.E., Takagi, T., Nakamura Y., Tsunoda, T.** (2001). Alterations of gene expression during colorectal carcinogenesis revealed by cDNA microarrays after laser-capture microdissection of tumor tissues and normal ephitelia. *Cancer Research.* **61**, 3544-49.
20. **Lassmann, S., Bauer, M., Rosenberg, R., Nekarda, H., Soong, R., Ruger, R., Hofler, H., Werner, M.** (2004). Identification of occult tumor cells in node negative lymph nodes of colorectal cancer patients by cytokeratin 20 gene and protein expression. *International Journal of Colorectal disease*. **19 (2)**. 87-94.
21. **Libra, M., Navolanic, P.M., Talamini, R., Cecchin, E., Sartor, F., Tumolo, S., Masier, S., Travali, S., Boiocchi, M., Toffoli, G.** (2004). Thymidylate synthase mRNA levels are increased in liver metasatases of colorectal cancer patients resistant to fluoropyrimidine-based chemotherapy. *BioMed Central Cancer.* **25 (4)**, 11.
22. **McGregor, D.K., Wu, T.T., Rashid, A., Luthra. R., Hamilton, S.R.** (2004). Reduced expression of cytokeratin 20 in colorectal carcinomas with high levels of microsatellite instability *American Journal of Surgical Pathology.* **28 (6),** 712-718.
23. **Notterman, D.A., Alon, U., Sierk, A.J., Levine, A.J.** (2001). Transcriptional gene expression profiles of colorectal adenoma, adrnocarcinoma, and normal tissue examined by oligonucleotide arrays. *Cancer Research.* **61**, 3124-30.
24. **Oberg, A.N., Lindmark, G.E., Israelsson, A.C., Hammarstrom, S.G., Hammarstrom, M.L.** (2004). Detection of occult tumor cells in lymph nodes of colorectal cancer patients using real-time quantitative RT-PCR for CEA and CK20 mRNAs. *International Journal of Cancer.* **111 (1)**, 101-10.
25. **Popat, S., Matakidou, A., Houlston, R.S.** (2004). Thymidylate synthase expression and prognosis in colorectal cancer: a systematic review and meta-analysis. *Journal of Clinical Oncology.* **22 (3)**, 529-36.
26. **Robertson, N., Potter, C., Harris, A.L.** (2004). Role of carbonic anhydrase IX in human tumor cell growth, survival, and invasion. *Cancer Research.* **64 (17)**, 6160-5.
27. [**Sakakibara, T**](http://www.ncbi.nlm.nih.gov/entrez/query.fcgi?db=pubmed&cmd=Search&itool=pubmed_Abstract&term="Sakakibara+T"%5BAuthor%5D)., [**Hibi, K**](http://www.ncbi.nlm.nih.gov/entrez/query.fcgi?db=pubmed&cmd=Search&itool=pubmed_Abstract&term="Hibi+K"%5BAuthor%5D)., [**Koike, M**](http://www.ncbi.nlm.nih.gov/entrez/query.fcgi?db=pubmed&cmd=Search&itool=pubmed_Abstract&term="Koike+M"%5BAuthor%5D)., [**Fujiwara, M**](http://www.ncbi.nlm.nih.gov/entrez/query.fcgi?db=pubmed&cmd=Search&itool=pubmed_Abstract&term="Fujiwara+M"%5BAuthor%5D)., [**Kodera, Y**](http://www.ncbi.nlm.nih.gov/entrez/query.fcgi?db=pubmed&cmd=Search&itool=pubmed_Abstract&term="Kodera+Y"%5BAuthor%5D)., [**Ito, K**](http://www.ncbi.nlm.nih.gov/entrez/query.fcgi?db=pubmed&cmd=Search&itool=pubmed_Abstract&term="Ito+K"%5BAuthor%5D)., [**Nakao, A**](http://www.ncbi.nlm.nih.gov/entrez/query.fcgi?db=pubmed&cmd=Search&itool=pubmed_Abstract&term="Nakao+A"%5BAuthor%5D). (2005). Plasminogen activator inhibitor-1 as a potential marker for the malignancy of colorectal cancer. *Br. J. Cancer.* **93(7),** 799-803.
28. **Sela, B.** (2002). Survivin: anti-apoptosis protein and a prognostic marker for tumor progression and recurrence. *Harefuah.* **141 (1)**, 103-7.
29. **Soumaoro, L.T., Uetake, H., Higuchi, T., Takagi, Y., Enomoto, M., Sugihara, K.** (2004). Cyclooxygenase-2 expression: a significant prognostic indicator for patients with colorectal cancer. *Clinical Cancer Research.* **10**, 8465-71.
30. **Suga, K., Yamamoto, T., Yamada, Y., Miyatake, S-I., Nakagawa, T., Tanigawa, N.** (2005). Correlation between transcriptional expression of survivin isoforms and clinicopathological findings in human colorectal carcinomas. *Oncology Reports.* **13**, 891-97.
31. **Terada, H., Urano, T., Cono, H.** (2005). Association of Interleukin-8 and plasminogen activator system in the progresión of colorectal cancer. *Eur Surg Res*. **37 (3)** 166-72.
32. **Valbohmer, D., Kuramochi, H., Shimizu, D., Danenberg, K.D., Lindebjerg, J., Nielsen, J.N., Jakobsen, A., Danenberg, P.V.** (2006). Molecular factors of 5-fluorouracil metabolism in colorectal cancer: Analysis of primary tumor and lymph node metastasis. *International Journal of Oncology.* **28 (2)**, 527-33.
33. **Vizoso, F.J., Fernandez, J.C., Corte, M.D., Bongera, M., Gava, R., Allende, M.T., Garcia-Muniz, J.L., Garcia-Moran, M.** (2004). Expression and clinical significance of CD44v5 and CD44v6 in resectable colorectal cancer. *J. Cancer Res. Clin. Oncol.* **130 (11),** 679-86.
34. **White, J.F., Hewett, P.W., Kosuge, D., McCulloch, T., Rnholm, B.C., Carmichael, J., Murray, J.C.** (2002). Vascular endothelial growth factor-D expression is an independent prognostic marker for survival in colorectal carcinoma. *Cancer Research*. **62**, 1669-75.
35. **Yao, H.B., Wu, A.G., Cheng, Y.J., Tang, B.H.** (2005). Expression of COX-2 protein in colorectal carcinoma and the clinical implication. *Di Yi Jun Yi Da Xue Xue Bao.* **25 (12)**, 1524-28.
36. **Yao, H.B., Wu, A.G., Huang, Z.H.** (2004). Expression of survivin and its clinical significance in colorectal carcinoma. *Di Yi Jun Yi Da Xue Xue Bao.* **24 (12)**, 1412-15.
37. **Yeh, C.S., Wang, J.Y., Wu, C.H., Chong, I.W., Chung, F.Y., Wang, Y.H., Yu, Y.P., Lin, S.R.** (2006). Molecular detection of circulating cancer cells in the peripheral blood of patients with colorectal cancer by using membrane array with a multiple mRNA marker panel. *Int J Oncol.* **28 (2)**, 411-20.
38. **Zavrides, H.N., Zizi-Sermpetzoglou, A., Panausopoulos, D., Athanasas, G., Elemenoglou, I., Peros, G.** (2005). Prognostic evaluation of CD44 expression in correlation with bcl-2 and p53 in colorectal cancer. *Folia Histochem. Cytobiol.* **43 (1),** 31-36.
